# Phylogenetic network analysis of SARS-CoV-2 genomes

**DOI:** 10.1073/pnas.2004999117

**Published:** 2020-04-08

**Authors:** Peter Forster, Lucy Forster, Colin Renfrew, Michael Forster

**Affiliations:** ^a^Institute of Forensic Genetics, 48161 Münster, Germany;; ^b^McDonald Institute for Archaeological Research, University of Cambridge, Cambridge CB2 3ER, United Kingdom;; ^c^Fluxus Technology Limited, Colchester CO3 0NU, United Kingdom;; ^d^Lakeside Healthcare Group at Cedar House Surgery, St Neots PE19 1BQ, United Kingdom;; ^e^Institute of Clinical Molecular Biology, Christian-Albrecht-University of Kiel, 24105 Kiel, Germany

**Keywords:** SARS-CoV-2 evolution, subtype, ancestral type

## Abstract

In a phylogenetic network analysis of 160 complete human severe acute respiratory syndrome coronavirus 2 (SARS-Cov-2) genomes, we find three central variants distinguished by amino acid changes, which we have named A, B, and C, with A being the ancestral type according to the bat outgroup coronavirus. The A and C types are found in significant proportions outside East Asia, that is, in Europeans and Americans. In contrast, the B type is the most common type in East Asia, and its ancestral genome appears not to have spread outside East Asia without first mutating into derived B types, pointing to founder effects or immunological or environmental resistance against this type outside Asia. The network faithfully traces routes of infections for documented coronavirus disease 2019 (COVID-19) cases, indicating that phylogenetic networks can likewise be successfully used to help trace undocumented COVID-19 infection sources, which can then be quarantined to prevent recurrent spread of the disease worldwide.

## Significance

This is a phylogenetic network of SARS-CoV-2 genomes sampled from across the world. These genomes are closely related and under evolutionary selection in their human hosts, sometimes with parallel evolution events, that is, the same virus mutation emerges in two different human hosts. This makes character-based phylogenetic networks the method of choice for reconstructing their evolutionary paths and their ancestral genome in the human host. The network method has been used in around 10,000 phylogenetic studies of diverse organisms, and is mostly known for reconstructing the prehistoric population movements of humans and for ecological studies, but is less commonly employed in the field of virology.

The search for human origins seemed to take a step forward with the publication of the global human mitochondrial DNA tree (1). It soon turned out, however, that the tree-building method did not facilitate an unambiguous interpretation of the data. This motivated the development, in the early 1990s, of phylogenetic network methods which are capable of enabling the visualization of a multitude of optimal trees (2, 3). This network approach, based on mitochondrial and Y chromosomal data, allowed us to reconstruct the prehistoric population movements which colonized the planet (4, 5). The phylogenetic network approach from 2003 onward then found application in the reconstruction of language prehistory (6). It is now timely to apply the phylogenetic network approach to virological data to explore how this method can contribute to an understanding of coronavirus evolution.

**Fig. 1.**
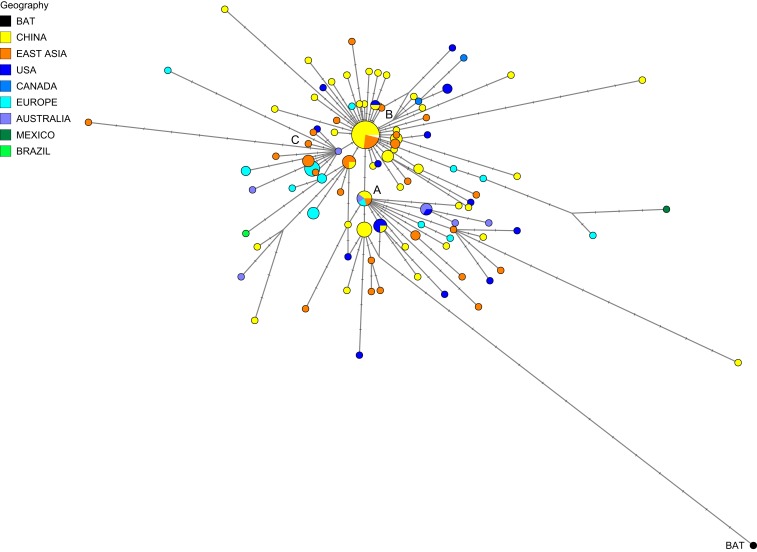
Phylogenetic network of 160 SARS-CoV-2 genomes. Node A is the root cluster obtained with the bat (*R. affinis*) coronavirus isolate BatCoVRaTG13 from Yunnan Province. Circle areas are proportional to the number of taxa, and each notch on the links represents a mutated nucleotide position. The sequence range under consideration is 56 to 29,797, with nucleotide position (np) numbering according to the Wuhan 1 reference sequence (8). The median-joining network algorithm (2) and the Steiner algorithm (9) were used, both implemented in the software package Network5011CS (https://www.fluxus-engineering.com/), with the parameter epsilon set to zero, generating this network containing 288 most-parsimonious trees of length 229 mutations. The reticulations are mainly caused by recurrent mutations at np11083. The 161 taxa (160 human viruses and one bat virus) yield 101 distinct genomic sequences. The phylogenetic diagram is available for detailed scrutiny in A0 poster format (*SI Appendix*, Fig. S5) and in the free Network download files.

In early March 2020, the GISAID database (https://www.gisaid.org/) contained a compilation of 253 severe acute respiratory syndrome coronavirus 2 (SARS-CoV-2) complete and partial genomes contributed by clinicians and researchers from across the world since December 2019. To understand the evolution of this virus within humans, and to assist in tracing infection pathways and designing preventive strategies, we here present a phylogenetic network of 160 largely complete SARS-Cov-2 genomes (Fig. 1).

Zhou et al. (7) recently reported a closely related bat coronavirus, with 96.2% sequence similarity to the human virus. We use this bat virus as an outgroup, resulting in the root of the network being placed in a cluster of lineages which we have labeled “A.” Overall, the network, as expected in an ongoing outbreak, shows ancestral viral genomes existing alongside their newly mutated daughter genomes.

There are two subclusters of A which are distinguished by the synonymous mutation T29095C. In the T-allele subcluster, four Chinese individuals (from the southern coastal Chinese province of Guangdong) carry the ancestral genome, while three Japanese and two American patients differ from it by a number of mutations. These American patients are reported to have had a history of residence in the presumed source of the outbreak in Wuhan. The C-allele subcluster sports relatively long mutational branches and includes five individuals from Wuhan, two of which are represented in the ancestral node, and eight other East Asians from China and adjacent countries. It is noteworthy that nearly half (15/33) of the types in this subcluster, however, are found outside East Asia, mainly in the United States and Australia.

Two derived network nodes are striking in terms of the number of individuals included in the nodal type and in mutational branches radiating from these nodes. We have labeled these phylogenetic clusters B and C.

For type B, all but 19 of the 93 type B genomes were sampled in Wuhan (*n* = 22), in other parts of eastern China (*n* = 31), and, sporadically, in adjacent Asian countries (*n* = 21). Outside of East Asia, 10 B-types were found in viral genomes from the United States and Canada, one in Mexico, four in France, two in Germany, and one each in Italy and Australia. Node B is derived from A by two mutations: the synonymous mutation T8782C and the nonsynonymous mutation C28144T changing a leucine to a serine. Cluster B is striking with regard to mutational branch lengths: While the ancestral B type is monopolized (26/26 genomes) by East Asians, every single (19/19) B-type genome outside of Asia has evolved mutations. This phenomenon does not appear to be due to the month-long time lag and concomitant mutation rate acting on the viral genome before it spread outside of China (Dataset S1, Supplementary Table 2). A complex founder scenario is one possibility, and a different explanation worth considering is that the ancestral Wuhan B-type virus is immunologically or environmentally adapted to a large section of the East Asian population, and may need to mutate to overcome resistance outside East Asia.

Type C differs from its parent type B by the nonsynonymous mutation G26144T which changes a glycine to a valine. In the dataset, this is the major European type (*n* = 11), with representatives in France, Italy, Sweden, and England, and in California and Brazil. It is absent in the mainland Chinese sample, but evident in Singapore (*n* = 5) and also found in Hong Kong, Taiwan, and South Korea.

One practical application of the phylogenetic network is to reconstruct infection paths where they are unknown and pose a public health risk. The following cases where the infection history is well documented may serve as illustrations (*SI Appendix*). On 25 February 2020, the first Brazilian was reported to have been infected following a visit to Italy, and the network algorithm reflects this with a mutational link between an Italian and his Brazilian viral genome in cluster C (*SI Appendix*, Fig. S1). In another case, a man from Ontario had traveled from Wuhan in central China to Guangdong in southern China and then returned to Canada, where he fell ill and was conclusively diagnosed with coronavirus disease 2019 (COVID-19) on 27 January 2020. In the phylogenetic network (*SI Appendix*, Fig. S2), his virus genome branches from a reconstructed ancestral node, with derived virus variants in Foshan and Shenzhen (both in Guangdong province), in agreement with his travel history. His virus genome now coexists with those of other infected North Americans (one Canadian and two Californians) who evidently share a common viral genealogy. The case of the single Mexican viral genome in the network is a documented infection diagnosed on 28 February 2020 in a Mexican traveler to Italy. Not only does the network confirm the Italian origin of the Mexican virus (*SI Appendix*, Fig. S3), but it also implies that this Italian virus derives from the first documented German infection on 27 January 2020 in an employee working for the Webasto company in Munich, who, in turn, had contracted the infection from a Chinese colleague in Shanghai who had received a visit by her parents from Wuhan. This viral journey from Wuhan to Mexico, lasting a month, is documented by 10 mutations in the phylogenetic network.

This viral network is a snapshot of the early stages of an epidemic before the phylogeny becomes obscured by subsequent migration and mutation. The question may be asked whether the rooting of the viral evolution can be achieved at this early stage by using the oldest available sampled genome as a root. As *SI Appendix*, Fig. S4 shows, however, the first virus genome that was sampled on 24 December 2019 already is distant from the root type according to the bat coronavirus outgroup rooting.

The described core mutations have been confirmed by a variety of contributing laboratories and sequencing platforms and can be considered reliable. The phylogeographic patterns in the network are potentially affected by distinctive migratory histories, founder events, and sample size. Nevertheless, it would be prudent to consider the possibility that mutational variants might modulate the clinical presentation and spread of the disease. The phylogenetic classification provided here may be used to rule out or confirm such effects when evaluating clinical and epidemiological outcomes of SARS-CoV-2 infection, and when designing treatment and, eventually, vaccines.

## Materials and Methods

The Global Initiative on Sharing Avian Influenza Data (GISAID) was founded in 2006, and, since 2010, has been hosted by the German Federal Ministry of Food, Agriculture and Consumer Protection. GISAID has also become a coronavirus repository since December 2019. As of 4 March 2020, the cutoff point for our phylogenetic analysis, the GISAID database (https://www.gisaid.org/) had compiled 254 coronavirus genomes, isolated from 244 humans, nine Chinese pangolins, and one bat *Rhinolophus affinis* (BatCoVRaTG13 from Yunnan Province, China). The sequences have been deposited by 82 laboratories listed in Dataset S1, Supplementary Table 1. Although SARS-CoV-2 is an RNA virus, the deposited sequences, by convention, are in DNA format. Our initial alignment confirmed an earlier report by Zhou et al. (7) that the pangolin coronavirus sequences are poorly conserved with respect to the human SARS-CoV-2 virus, while the bat coronavirus yielded a sequence similarity of 96.2% in our analysis, in agreement with the 96.2% published by Zhou et al. We discarded partial sequences, and used only the most complete genomes that we aligned to the full reference genome by Wu et al. (8) comprising 29,903 nucleotides. Finally, to ensure comparability, we truncated the flanks of all sequences to the consensus range 56 to 29,797, with nucleotide position numbering according to the Wuhan 1 reference sequence (8). The laboratory codes of the resulting 160 sequences and the bat coronavirus sequences are listed in Dataset S1, Supplementary Table 2 (Coronavirus Isolate Labels).

The 160 human coronavirus sequences comprised exactly 100 different types. We added to the data the bat coronavirus as an outgroup to determine the root within the phylogeny. Phylogenetic network analyses were performed with the Network 5011CS package, which includes, among other algorithms, the median joining network algorithm (3) and a Steiner tree algorithm to identify most-parsimonious trees within complex networks (9). We coded gaps of adjacent nucleotides as single deletion events (these deletions being rare, up to 24 nucleotides long, and mostly in the amino acid reading frame) and ran the data with the epsilon parameter set to zero, and performed an exploratory run by setting the epsilon parameter to 10. Both settings yielded a low-complexity network. The Steiner tree algorithm was then run on both networks and provided the identical result that the most-parsimonious trees within the network were of length 229 mutations. The structures of both networks were very similar, with the epsilon 10 setting providing an additional rectangle between the A and B clusters. The network output was annotated using the Network Publisher option to indicate geographic regions, sample collection times, and cluster nomenclature.

### Data Availability.

The nucleotide sequences of the SARS-CoV-2 genomes used in this analysis are available, upon free registration, from the GISAID database (https://www.gisaid.org/). The Network5011 software package and coronavirus network files are available as shareware on the Fluxus Technology website (https://www.fluxus-engineering.com/).

## Supplementary Material

Supplementary File

Supplementary File
