# Supplementary material for: Phylogenetic network analysis of SARS-CoV-2 genomes
Source: Proc Natl Acad Sci U S A. 2020 Apr 8;117(17):9241–3. doi: 10.1073/pnas.2004999117 (PMC7196762; doi:10.1073/pnas.2004999117)
Supplement: Supplementary File [file pnas.2004999117.sapp.pdf]

Supplementary Figures 1-5 with Figure Legends

Phylogenetic network analysis of SARS-CoV-2 genomes

Peter Forster[1,2,3], Lucy Forster[4], Colin Renfrew[2], Michael Forster[3,5]

[1] Institute of Forensic Genetics, 48161 Münster, Germany

[2] McDonald Institute for Archaeological Research, University of Cambridge, CB2 3ER, United Kingdom

[3] Fluxus Technology Limited, Essex, United Kingdom

[4] Lakeside Healthcare Group at Cedar House Surgery, St Neots, PE19 1BQ Cambridgeshire, United Kingdom

[5] Institute of Clinical Molecular Biology, Christian-Albrecht-University of Kiel, 24105 Kiel, Germany

Correspondence: Dr. Peter Forster, McDonald Institute for Archaeological Research, University of Cambridge, CB2 3ER, United Kingdom, email: pf223@cam.ac.uk, Tel. +44 1223 424035

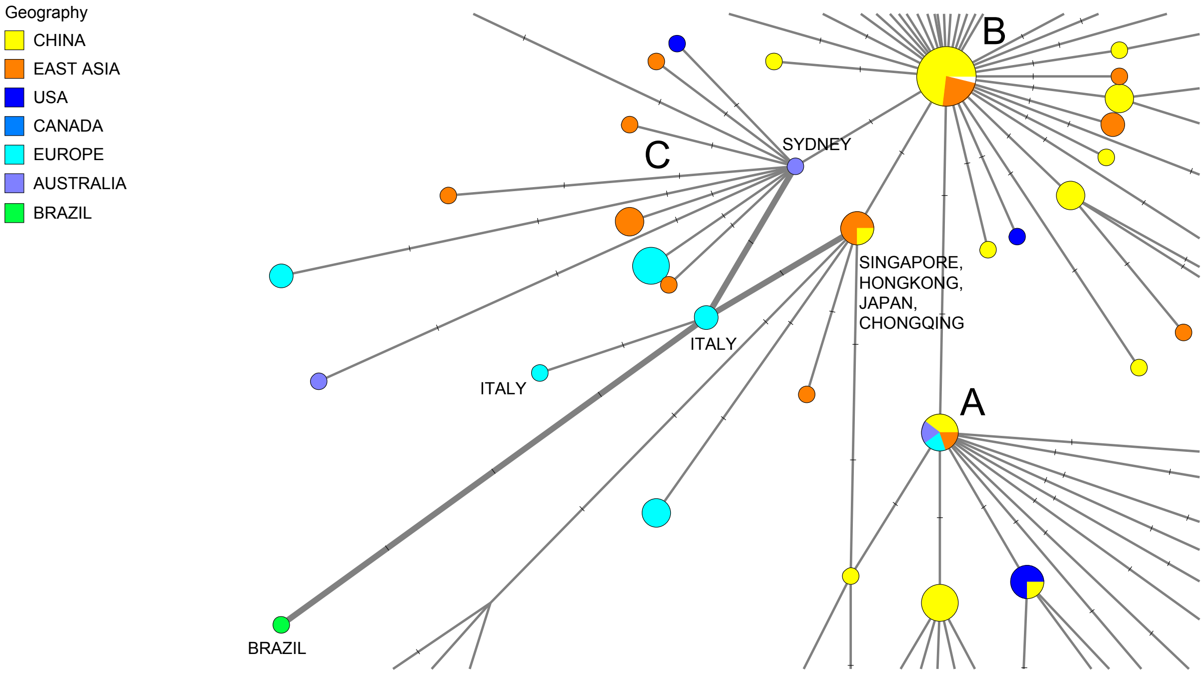

Supplementary Figure 1: Brazilian patient's virus located in phylogenetic network cluster C. The Brazilian patient had visited Italy before his diagnosis, and the network shows a mutational link between an Italian's and the Brazilian's viral genome. Detailed referenced case histories are available in the following dedicated Wikipedia article: https://en.wikipedia.org/wiki/2020_coronavirus_outbreak_in_Brazil

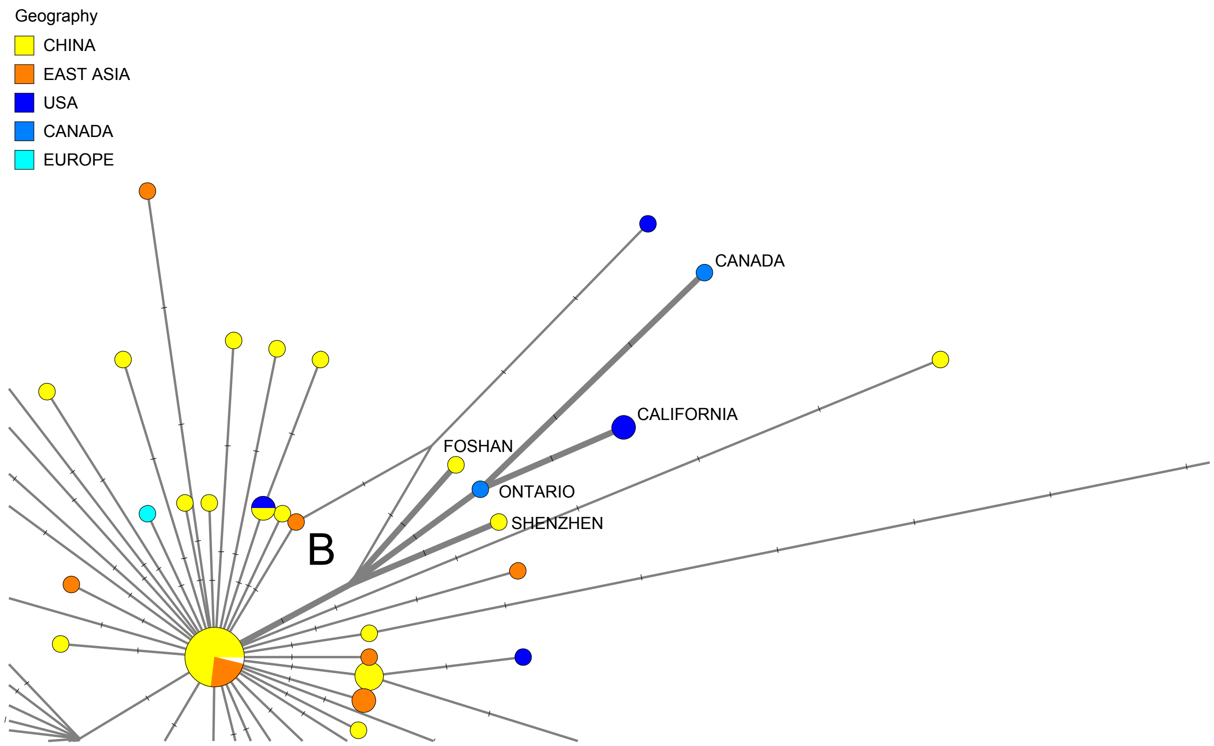

Supplementary Figure 2: Ontario patient's virus located in phylogenetic network cluster B. This patient had travelled from Wuhan in central China to Guangdong in southern China and then returned to Canada where he fell ill and was conclusively diagnosed with COVID-19 on January 27. His virus genome branches from a reconstructed ancestral node, with derived virus variants in Foshan and Shenzhen (both in Guangdong province), in agreement with his travel history. His virus genome now co-exists with those of other infected North Americans (one Canadian and two Californians). Detailed referenced case histories are available in the following dedicated Wikipedia articles: https://en.wikipedia.org/wiki/2020_coronavirus_outbreak_in_Canada, https://en.wikipedia.org/wiki/2020_coronavirus_outbreak_in_the_United_States.

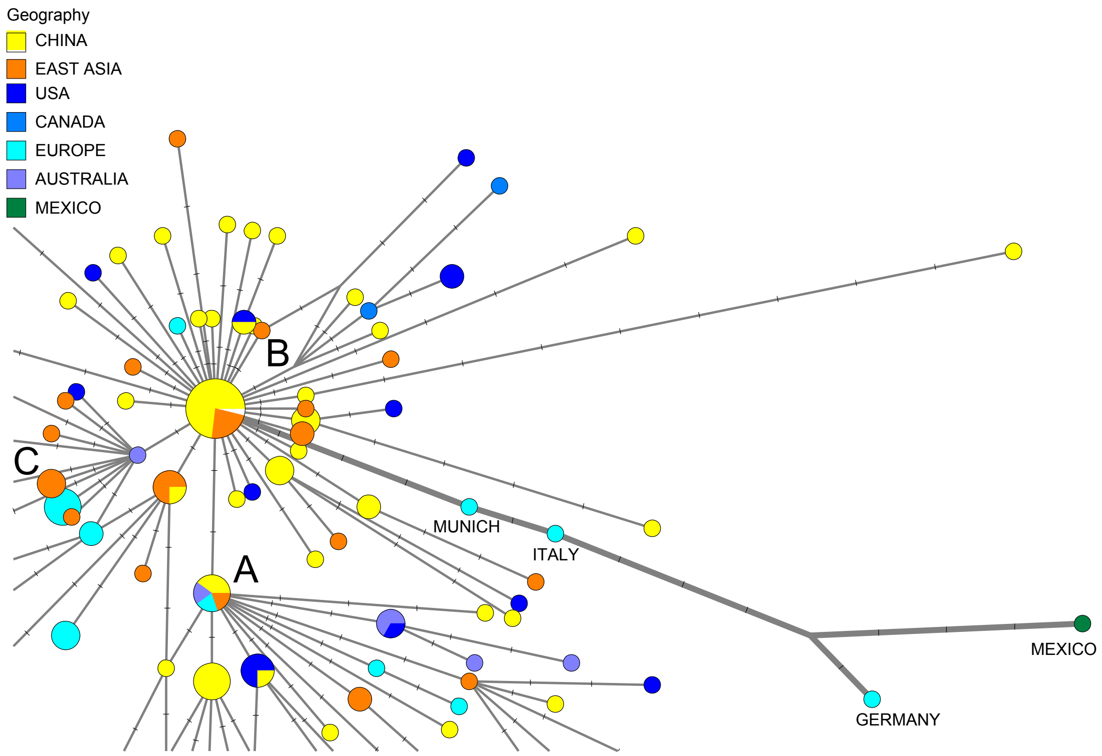

Supplementary Figure 3: Mexican patient's virus located in phylogenetic network cluster B. The Mexican patient had visited Italy before his diagnosis, and the network shows a mutational distance of 6 mutations between an Italian's and the Mexican's viral genome. The Italian's virus is linked to the Munich virus by one mutation's difference. The Munich virus has been extensively reported by the German media as the first German cluster of infections. Detailed referenced case histories are available in the following dedicated Wikipedia articles:
https://en.wikipedia.org/wiki/2020_coronavirus_outbreak_in_Mexico,
https://en.wikipedia.org/wiki/2020_coronavirus_outbreak_in_Germany

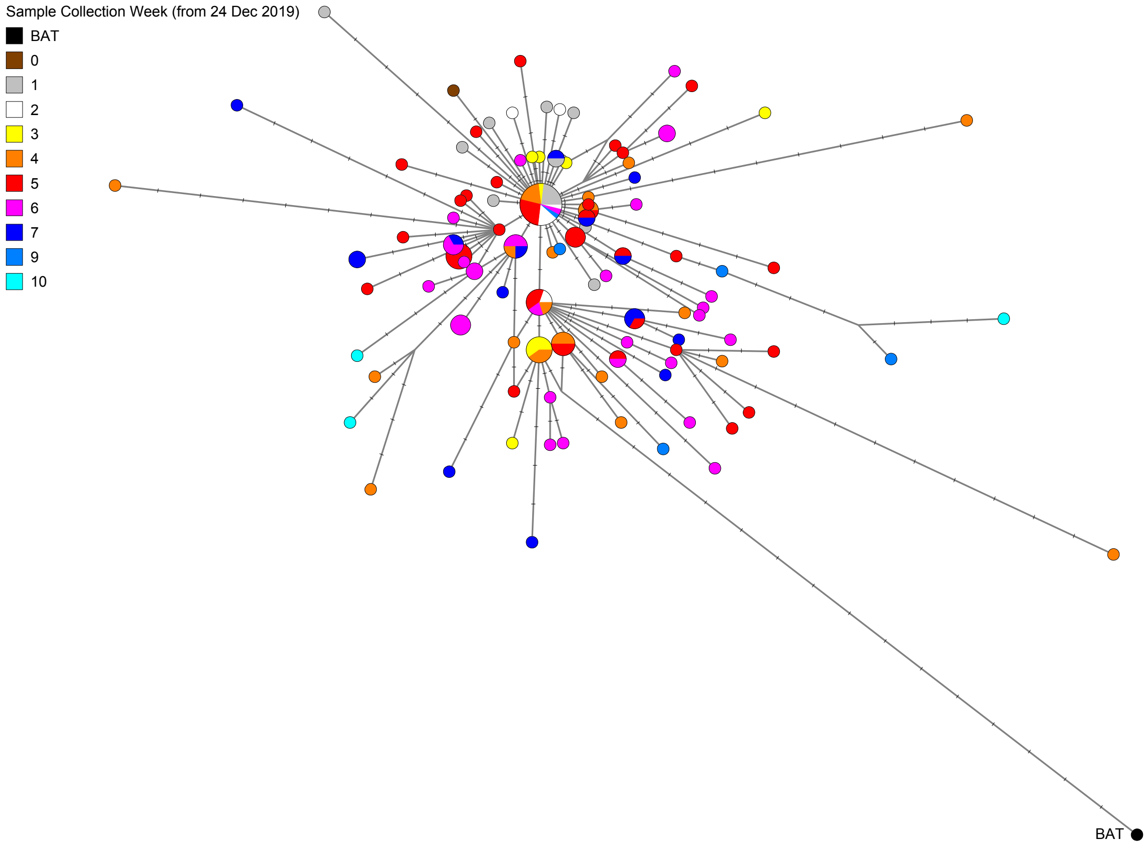

Supplementary Figure 4: Phylogenetic network indicating the week of sample collection. According to the GISAID SARS-CoV-2 database, the first virus isolate was collected on 24 December 2019, hence week zero is counted from 24-30 December 2019. The sample collection date for two Italian isolates was given as "January 2020" (which we then averaged as 15 January) and for all remaining isolates the exact day was given. Note that the oldest isolate from 24 December 2019 (brown node, week 0) lies diagonally opposite to the bat virus outgroup root.

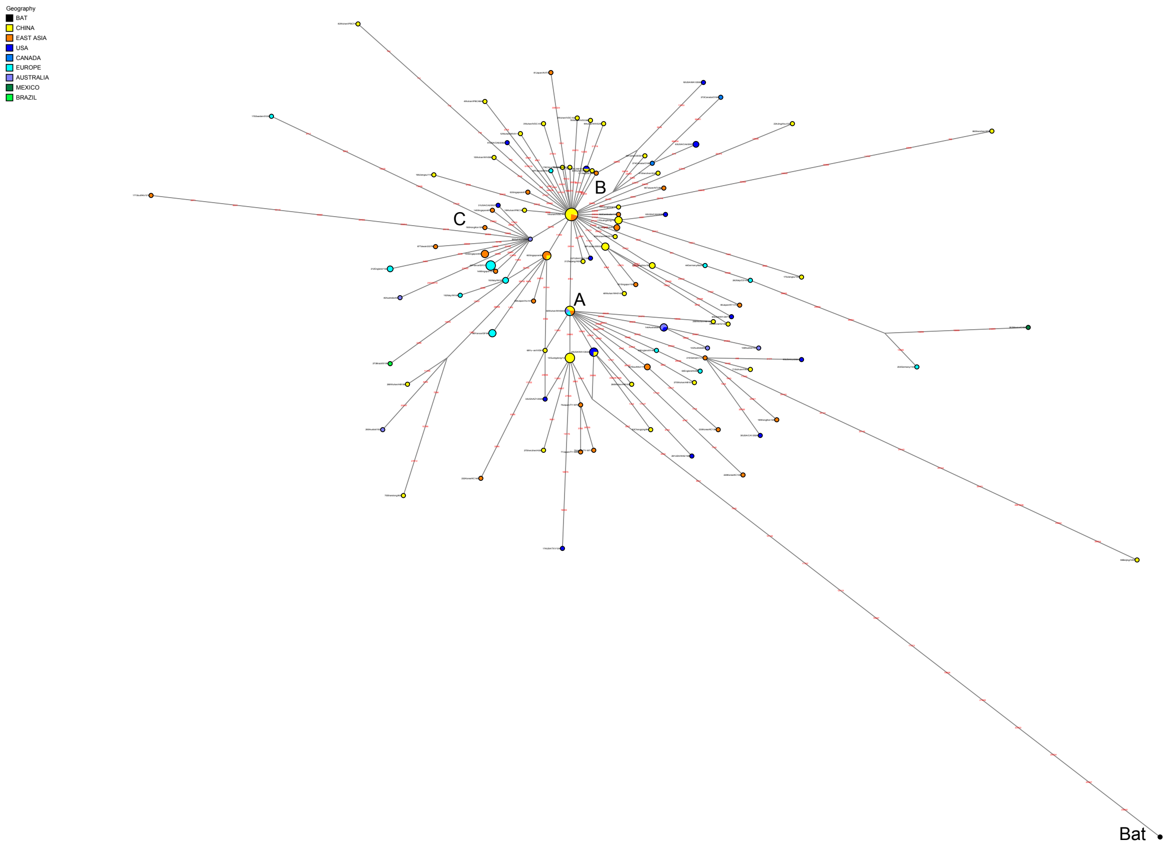

Supplementary Figure 5: Detailed phylogenetic network showing the nodes labelled with virus genome names and the links labelled with the mutated positions (except for the in-frame deletion stretches which have been replaced by a single position, see Methods). The Figure is formatted for A0-size or for interactive use in a PDF viewer. Network nodes that comprise multiple virus genome names are labelled with one representative name. For the complete list of names in a multi-virus-node please consult the Network files in a text editor or in Network Publisher.
